# How do perceived design factors relate to user evaluations of multicultural commercial streets? Evidence from three sites in South Korea

**DOI:** 10.1371/journal.pone.0358048

**Published:** 2026-10-05

**Authors:** Duanduan Liu, Fan Xu, Taesun Kim

**Affiliations:** 1 Department of Industrial Design, ERICA Campus, Hanyang University, Ansan, Korea; 2 Robotics Institute, Ningbo University of Technology, Ningbo, China; Gachon University, KOREA, REPUBLIC OF

## Abstract

Evaluating the design of multicultural commercial streets extends beyond assessing cultural symbols; it also requires understanding how users perceive place through the physical environment, cultural symbolism, and digital communication. This study examined the Ansan Multicultural Special Zone, Daerim Central Market in Seoul, and Incheon Chinatown. A theory-guided observed-variable path model was developed using five perceived design factors: Convenience (CO), Social Recognition and Sharing (SO), Aesthetic (AE), Symbolism (SY), and Trendiness (TR). All 142 participants evaluated all three sites. Data were analyzed using descriptive statistics, Pearson correlations, repeated-measures ANOVA, site-specific path models, and linear mixed-effects models. Incheon Chinatown received higher ratings on most factors. Five paths—AE → SY, AE → TR, AE → CO, SY → TR, and TR→SO—were significant at all three sites, with no significant cross-site differences. The SY→SO path was not significant in Ansan, suggesting that cultural symbolism was not directly associated with ratings of Social Recognition and Sharing at this policy-designated site. SO→CO showed evidence of cross-site variation, with the strongest association observed at Daerim Central Market in Seoul. These findings indicate that evaluations of multicultural commercial streets reflect both differences in mean ratings and structural associations among visual quality, cultural symbolism, online visibility, social recognition and sharing, and functional convenience. These findings highlight the value of integrating cultural representation with visual quality, online visibility, social recognition and sharing, and ease of use in regeneration strategies.

## Introduction

As globalization has intensified, cross-border migration and cultural coexistence have become increasingly prominent. These demographic and cultural changes have created new demands for urban spaces and contributed to distinctive multicultural urban characteristics. Florida [1] links openness to diversity with cities’ capacity to attract creative talent and compete economically. Sohrabi [2] notes that multiculturalism has also prompted debates about social cohesion and related tensions. Hassen and Giovanardi [3] show how ethnic diversity can be incorporated into city branding through openness, inclusivity, services for foreign businesses, and opportunities for cultural expression. Niebuhr [4] suggests that differences in knowledge, experience, and skills among culturally diverse groups may serve as important resources for regional innovation. Fainstein [5] identifies recognition of diversity as a central concern in debates about urban justice. Hunter [6] demonstrates that online visual representations contribute to the social construction of Seoul’s tourism destination image. Together, these studies link cultural diversity to urban competitiveness, identity, planning, and mediated representations of place. Multicultural urban design is therefore associated with economic vitality, openness, and city branding, while also presenting new challenges for urban planners.

South Korea has experienced substantial growth in its immigrant population since the 1980s, particularly among people entering the country for marriage or employment [7]. As of January 1, 2026, Ansan had 101,200 foreign residents within a total population of 713,708 [8]. Wongok-dong was designated as the Ansan Multicultural Village Special Zone in 2009 [9]. In Seoul, Daerim-dong has developed into a major Korean-Chinese enclave in which migrant businesses, everyday services, and community life are closely intertwined [10]. Incheon Chinatown developed from the historical Chinese settlement associated with the opening of Incheon Port and is now characterized by the spatial representation of Chinese cultural heritage and tourism-oriented commercial activities [11].

However, evaluations of multicultural commercial street design involve more than cultural diversity alone. Research on urban design and servicescapes has linked accessibility, spatial interfaces, and streetscape quality to users’ evaluations of commercial environments. Similarly, studies of place image and digital communication have linked cultural representation and online information to the formation and circulation of place perceptions [12–16]. Existing studies have primarily examined multicultural spaces through the lenses of city branding, multicultural governance, public attitudes toward immigration policy, and urban citizenship [3,9,17,18]. Nevertheless, systematic comparative research on how users perceive specific design factors and how these factors jointly relate to the place image of multicultural commercial streets remains limited.

To address this gap, the present study examines three multicultural commercial streets in South Korea: the Ansan Multicultural Special Zone, Daerim Central Market in Seoul, and Incheon Chinatown. These sites represent three distinct types of multicultural commercial districts: a policy-designated district, an everyday transnational commercial district, and a historical tourism district. Together, they reflect distinct developmental trajectories among multicultural commercial streets in South Korea. Drawing on research on urban design, place image, destination image, and digital communication, this study identifies five perceived design factors: Convenience (CO), Social Recognition and Sharing (SO), Aesthetic (AE), Symbolism (SY), and Trendiness (TR). These factors were modeled as observed variables. A theory-guided observed-variable path model was then developed to compare the three sites, examine associations among the five factors, and test whether path relationships varied across site contexts. The study focuses on two dimensions of design evaluation: differences in perceived design factor ratings across sites and structural associations among the physical environment, cultural symbolism, digital communication, social recognition and sharing, and functional convenience. Identifying cross-site variation in path strength may also help determine whether multicultural commercial streets with different developmental backgrounds require tailored regeneration strategies and design-management approaches.

This study addresses the following research questions

RQ1: How do users’ ratings of the five perceived design factors—CO, SO, AE, SY, and TR—vary across the three multicultural commercial streets?

RQ2: To what extent does the empirical evidence from each study site support the seven prespecified theoretical paths among the five perceived design factors?

RQ3: Do the strengths of these prespecified paths vary across the three study sites, and which paths show site-specific variation?

### Theoretical background and research framework

#### Theoretical sources and operationalization of the perceived design factors.

Evaluations of multicultural commercial streets encompass not only commercial functions and cultural diversity but also users’ overall judgments of the spatial environment, cultural symbols, and the districts’ visibility through communication channels. Bitner’s servicescape theory [19] describes how the physical environment relates to users’ cognitive, emotional, and behavioral responses to service spaces. Turley and Milliman [20] further classify the external interfaces, interior design, layout, color, lighting, and atmosphere of commercial spaces as environmental variables relevant to consumer evaluations and behavior. Lee and Park’s streetscape study [21] likewise links the visual street environment and spatial interface characteristics to pedestrians’ visual perceptions and perceived walkability.

Beyond the physical environment, multicultural commercial streets are also shaped by cultural representation and social communication. Mehta and Bosson [22] highlight the role of street environments in supporting social life, while Echtner and Ritchie [12] conceptualize destination image as an integrated structure comprising attribute-based cognition, overall impressions, and recognition of uniqueness. Kavaratzis [13] further notes that city image is constituted not only by physical space but also through symbols, identity, and communication processes. Similarly, Xiang and Gretzel [14] identify social media as an important source through which tourists obtain place information and form destination perceptions. Lam et al. [15] and Guerreiro et al. [16] also show that user-generated content and online platforms contribute to the dissemination and reproduction of place image.

Drawing on this literature, this study defines five perceived design factors: Convenience (CO), Social Recognition and Sharing (SO), Aesthetic (AE), Symbolism (SY), and Trendiness (TR). CO captures the district’s accessibility and ease of use; SO, termed Social Recognition and Sharing in this study, captures the perceived extent to which the district is known and shared through social networking; AE captures the visual quality of the streetscape and spatial interfaces; SY captures cultural symbols and place representation; and TR captures the district’s visibility on online platforms. The subsequent analyses treat these five factors as observed variables.

### Theory-guided observed-variable path model

Using the five perceived design factors, this study develops a theory-guided observed-variable path model. The model treats AE as the starting point for physical and visual evaluation and CO as the endpoint representing functional convenience, while SY, TR, and SO describe the associative process through which cultural recognition, online visibility, and social recognition and sharing operate in evaluations of multicultural commercial streets. The seven paths fall into three theoretical categories: direct paths originating from AE, communication-transformation paths originating from SY, and cognitive associations among TR, SO, and CO.

The first category comprises paths originating from AE. Bitner [19] and Turley and Milliman [20] both link the physical environment and atmospheric cues in commercial spaces to user evaluations. Lee and Park [21] likewise associate visual street quality and spatial interface characteristics with pedestrians’ environmental perceptions. Accordingly, this study treats AE as the core visual design factor in evaluations of multicultural commercial streets. Higher visual quality is generally associated with clearer recognition of cultural characteristics, greater spatial legibility, and stronger perceptions of convenience. The model therefore specifies positive AE → SY and AE → CO paths. Xiang and Gretzel [14], Lam et al. [15], and Guerreiro et al. [16] also emphasize the role of social media and user-generated content in communicating place image. Districts that are more visually appealing and photogenic may be more likely to generate shareable visual content [6,14–16]; therefore, the model specifies a positive AE → TR path.

The second category comprises paths originating from SY. Echtner and Ritchie [12] conceptualize destination image as an integrated structure comprising attribute-based cognition, overall impressions, and recognition of uniqueness. Kavaratzis [13] further notes that city image is shaped and communicated through space, symbols, identity, and communication processes. Drawing on this literature, districts with distinct cultural symbols, landmarks, and representative spatial characteristics are more likely to develop a recognizable place image, which may in turn provide content for online communication [14 –16]. Similarly, recognizable cultural symbols and representative spatial characteristics may provide distinctive place-related content that users can recognize and share through social networking [13 –16]. The model therefore specifies positive SY → TR and SY→SO paths.

The third category concerns the relationships among TR, SO, and CO. Xiang and Gretzel [14] argue that social media plays an important role in place-information searches and destination perceptions. Lam et al. [15] and Guerreiro et al. [16] further indicate that online platforms and user-generated content contribute to the co-construction and diffusion of place image. Thus, greater online visibility may be associated with higher levels of recognition and sharing through social networking; accordingly, the model specifies a positive TR→SO path. Place information encountered and shared through social networks may increase users’ familiarity with a district’s location, access routes, store distribution, and activities [14 –16]. Such familiarity may, in turn, support more favorable perceptions of Convenience; accordingly, the model specifies a positive SO→CO path. Fig 1 summarizes the theoretical development of the five perceived design factors and their organization within the proposed path model.

**Fig 1:**
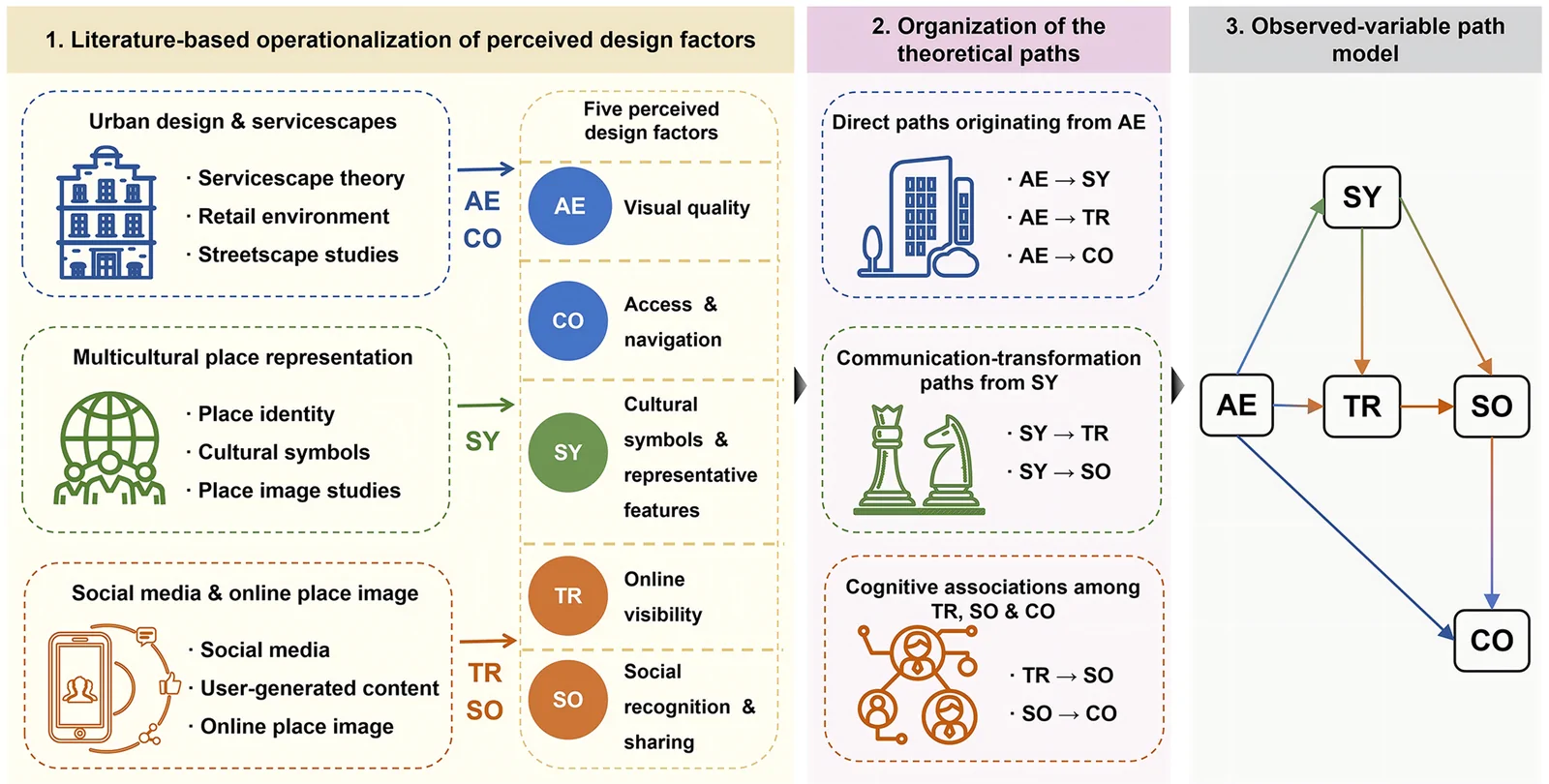
Theoretical development and operationalization of the five perceived design factors and the observed-variable path model.

## Methods

### Study Site Selection

Comparing multicultural commercial streets can reveal differences in perceived design factor ratings and their patterns of association. Because multicultural commercial streets develop within distinct national institutional, urban policy, immigration, and local commercial contexts, we used a within-country, multiple-case comparative design to reduce the influence of cross-national contextual differences on the interpretation of the results. Case studies are well suited to examining complex spatial phenomena in real-world settings, and case selection should enable theoretical comparison and differentiation among types [23,24].

Table 1 presents the three selected study sites: the Ansan Multicultural Special Zone, Daerim Central Market, Seoul, and Incheon Chinatown. All three sites have clearly defined multicultural contexts and comprise commercial streets, markets, or tourism-oriented commercial districts that users can directly perceive and evaluate. They also represent distinct types of multicultural commercial streets in South Korea. The Ansan Multicultural Special Zone is a policy-designated multicultural village special zone [25]. Daerim Central Market, Seoul, is a transnational commercial district centered on Korean Chinese immigrant businesses and everyday services [26]. Incheon Chinatown is a Chinese cultural district characterized by its historical development, symbolic landscapes, and tourism-oriented functions, with its special-zone boundaries and policy status documented in relevant public sources and previous studies [27,28]. More broadly, research on Chinatowns has shown how ethnic identities can be constructed and communicated through tourism discourse [29]. Thus, the three sites have distinct developmental backgrounds, represent major types of multicultural commercial streets in South Korea, and are suitable for comparative analysis.

**Table 1:** Comparison of the key characteristics of the three study sites.

Characteristic	Ansan Multicultural Special Zone	Daerim Central Market, Seoul	Incheon Chinatown
Representative type	Policy-designated type	Everyday transnational commercial type	Historical tourism type
Formation background	Designated as a multicultural village special zone in 2009 [25]	Gradually developed since the 2000s [26]	Gradually developed after 1884 [28]
Spatial extent	405,918.4 m² [25]	The market and surrounding commercial streets [26]	114,316 m² [27]
Multicultural basis	Foreign residents and foreign-national compatriots accounted for 89.2% of the population within the special zone [25].	Concentration of Korean Chinese immigrant businesses [26]	Chinese historical culture and commercial district [28]
Spatial character	Multicultural food street / mixed residential and commercial district [25]	Market / dining / everyday services [26]	Chinatown / historical culture and tourism consumption [27,28]
Policy or function	Multicultural village special zone [25]	Transnational commercial and community living space [26]	Regional specialized development zone [27]

From March 3 to April 10, 2023, the research team conducted field visits to the three selected sites: Wongok-dong within the Ansan Multicultural Special Zone, Daerim Central Market in Yeongdeungpo-gu, Seoul, and Chinatown in Jung-gu, Incheon. These visits coincided with the questionnaire data-collection period and served to verify the spatial settings and commercial functions of the selected districts, document their representative streetscape, commercial, and cultural characteristics, and provide the setting for the on-site component of the survey, through which 51 valid questionnaires were collected. Fig 2 provides visual documentation of the spatial, commercial, and cultural contexts of the three study sites during these field visits.

**Fig 2:**
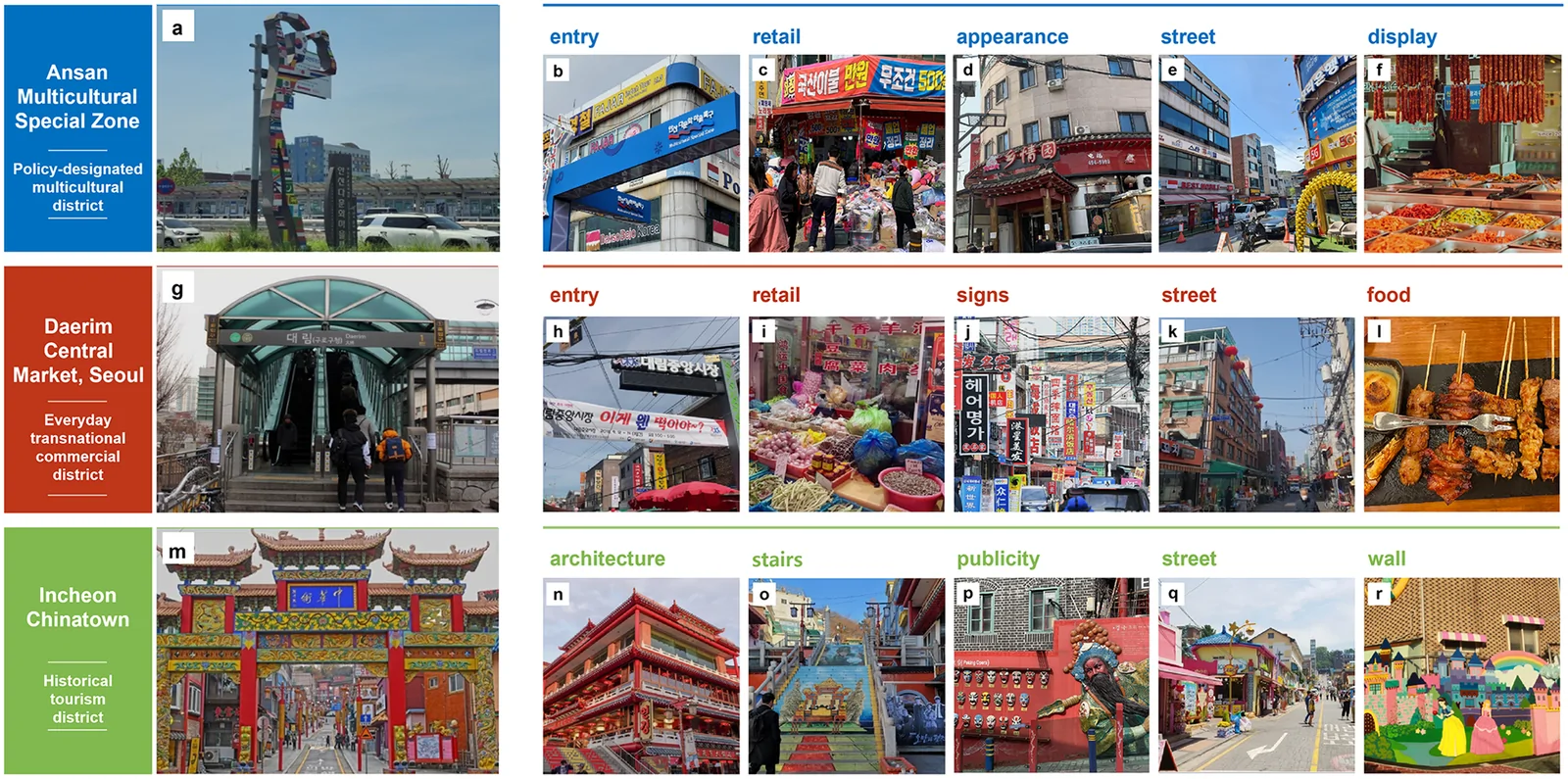
Field documentation of the three study sites. (a–f) Ansan Multicultural Special Zone; (g–l) Daerim Central Market, Seoul; (m–r) Incheon Chinatown. Photographs were taken from March 3 to April 10, 2023.

### Questionnaire design and data collection

The questionnaire measured the five perceived design factors. As shown in Table 2, each factor was assessed with a single overall item. Previous measurement studies have shown that well-defined single-item measures can reduce respondent burden while maintaining some predictive validity when respondents directly assess clearly defined targets [30,31]. Because each participant evaluated three study sites consecutively, this approach helped reduce the burden of repeated evaluations. The complete questionnaire is provided in S1 Text.

**Table 2:** Operationalization of the five perceived design factors.

Factor (code)	Operational focus	Survey item
Convenience (CO)	Access and navigation	Degree to which the multicultural zone is easy to access and navigate.
Social Recognition and Sharing (SO)	Recognition and sharing through social networks	Extent to which the area is known and shared through social networking.
Aesthetic (AE)	Visual quality	Aesthetic level of street landscapes and architectural design.
Symbolism (SY)	Cultural symbols and representative features	Presence of buildings or areas with distinct representativeness or symbolic significance.
Trendiness (TR)	Online visibility	Extent to which the area has become an internet hotspot or viral street.

Before the formal survey, 20 independent participants completed a pretest. The pretest assessed item wording, content comprehension, and the response process, and several phrases were revised in response to their feedback. Questionnaire pretesting can identify potential problems with item comprehension, response processes, and completion procedures [32]. Pretest participants were excluded from the final sample.

The formal questionnaire survey was conducted from March 3 to April 10, 2023, and focused on the Ansan Multicultural Special Zone, Daerim Central Market, Seoul, and Incheon Chinatown. Eligible participants were at least 18 years old and were expected to have prior experience of all three study sites. Familiarity was not defined using a specific threshold or separate screening procedure. We used convenience sampling. Of the 210 questionnaires distributed, 142 valid responses were obtained, for a valid response rate of 67.6%. Among these responses, 51 were collected on site and 91 online. Online participants were recruited primarily through local communities and social media groups.

Each participant evaluated all three study sites, yielding 142 sets of evaluations per site. The site-specific correlation analyses and path models therefore used 142 evaluations for each site. For cross-site comparisons, the data were reorganized into long format, yielding 426 site-level observations. Repeated-measures ANOVA and linear mixed-effects models accounted for within-person correlations.

Questionnaires were collected anonymously. Formal ethical review was considered unnecessary based on the exemption criteria under Article 13 of the Enforcement Rule of the Korean Bioethics and Safety Act, because no personally identifiable or sensitive information was collected and no vulnerable populations were involved. Before completing the questionnaire, participants read information about the study and provided informed consent. Online participants indicated consent by checking a box, whereas on-site participants provided written confirmation.

### Data analysis

We used SAS 9.4 for all analyses. Because each participant evaluated the Ansan Multicultural Special Zone, Daerim Central Market, Seoul, and Incheon Chinatown, the data included both cross-site comparisons and within-person repeated measures. The analyses were therefore organized around the three research questions.

To address RQ1, we calculated the means, standard deviations, and Pearson correlation coefficients for CO, SO, AE, SY, and TR to describe the distributions and linear associations of the five perceived design factors within each site. We then used repeated-measures ANOVA to compare the mean values of the five perceived design factors across the three sites. Sphericity was assessed using Mauchly’s test. When this assumption was violated, we reported Greenhouse–Geisser-corrected degrees of freedom and p values. Significant omnibus effects were followed by Bonferroni-adjusted pairwise comparisons; effect sizes were reported as partial η².

To address RQ2, we separately estimated observed-variable path models with the same structure for the three study sites, using the path structure proposed in the preceding theory-guided observed-variable path model. Path analysis estimated directional associations among observed variables within a theoretically prespecified structure and evaluated the fit of the model to the sample data [33,34]. The unified model comprised seven paths: AE → SY, AE → TR, SY → TR, SY→SO, TR→SO, AE → CO, and SO→CO. Overall model fit was evaluated using χ², CFI, TLI, RMSEA, and SRMR.

To address RQ3, we used linear mixed-effects models to test whether the same paths varied across study sites. Following the component-model approach to piecewise structural equation modeling, we decomposed the unified path structure into four regression equations with SY, TR, SO, and CO as dependent variables [35]. Each model included participant ID as a random intercept to account for within-person correlations arising from the same participant’s repeated evaluations of the three sites [36]. We estimated the linear mixed-effects models using REML and calculated denominator degrees of freedom for fixed-effect tests using the Satterthwaite approximation. Continuous predictors were mean-centered before constructing the interaction terms, and variance inflation factors (VIFs) were calculated from the fixed-effects design matrix to assess multicollinearity [37]. Each model included the study site, corresponding predictor variables, and “predictor × study site” interaction terms. These interaction terms tested whether path relationships differed across sites. The seven interaction tests corresponded to the seven prespecified path-specific hypotheses and were evaluated separately; therefore, their p values are reported without multiplicity adjustment. For an interaction with an unadjusted p value below .05, we calculated simple slopes and pairwise slope differences. The three pairwise slope comparisons were adjusted using the Holm procedure.

As a supplementary diagnostic, we conducted Harman’s single-factor test using all 15 site-specific observed variables to provide a preliminary assessment of the risk of common method bias [38]. Because the SO→CO interaction was the only one meeting this unadjusted .05 threshold, we also estimated a supplementary linear mixed-effects model that included gender, age, education, and survey mode as control variables to assess the robustness of this difference. Fig 3 summarizes the overall research workflow and analytical procedure.

**Fig 3:**
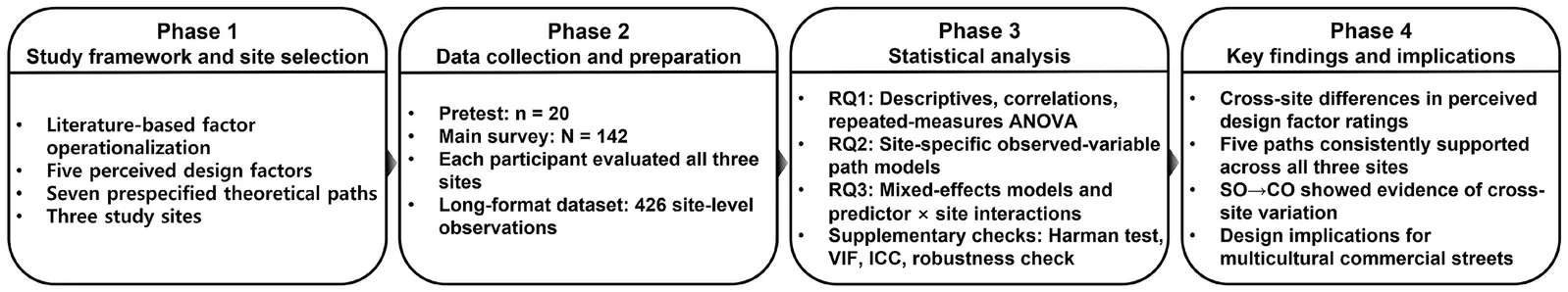
Overall research workflow and analytical procedure.

## Results

### Participant characteristics

Participant characteristics are summarized in Table 3. Among the 142 participants, 69 (48.6%) were aged 25–29 years, 83 (58.5%) were female, and 131 (92.3%) had a university degree or higher.

**Table 3:** Participant characteristics (N = 142).

Items		On-site	Online	Total
Age	18-24	8	30	38 (26.8%)
	25-29	36	33	69 (48.6%)
	30-34	5	17	22 (15.5%)
	35-40	2	11	13 (9.2%)
	Total	51	91	142 (100%)
Gender	Male	38	21	59 (41.5%)
	Female	13	70	83 (58.5%)
	Total	51	91	142 (100%)
Education level	High School	1	10	11 (7.8%)
	University degree	17	52	69 (48.6%)
	Graduate degree or above	33	29	62 (43.7%)
	Total	51	91	142 (100%)

### Descriptive statistics and correlation analysis

The descriptive statistics and Pearson correlations for the five perceived design factors within each study site are presented in (Table 4).

**Table 4:** Descriptive statistics and Pearson correlations for the three study sites (N = 142).

Factor	Site	CO	SO	AE	SY	TR	M ± SD
CO	Ansan	1.00					3.69 ± 0.97
	Seoul	1.00					3.71 ± 1.04
	Incheon	1.00					3.99 ± 0.84
SO	Ansan	.390	1.00				3.46 ± 1.04
	Seoul	.536	1.00				3.66 ± 1.03
	Incheon	.372	1.00				3.72 ± 0.98
AE	Ansan	.508	.276	1.00			3.30 ± 1.11
	Seoul	.397	.360	1.00			3.35 ± 1.09
	Incheon	.472	.438	1.00			3.96 ± 0.96
SY	Ansan	.393	.372	.645	1.00		3.44 ± 1.04
	Seoul	.438	.468	.571	1.00		3.35 ± 0.95
	Incheon	.315	.501	.515	1.00		3.99 ± 0.83
TR	Ansan	.398	.428	.636	.613	1.00	3.33 ± 1.10
	Seoul	.471	.479	.572	.643	1.00	3.47 ± 0.99
	Incheon	.367	.532	.385	.488	1.00	3.92 ± 0.89

Note: Only lower-triangle correlations are shown. All correlation coefficients were statistically significant at p ≤ .001.

All five perceived design factors were significantly and positively correlated at each study site. In the Ansan Multicultural Special Zone, Aesthetic showed the strongest correlation with Symbolism (r = .645, p < .001), indicating a close association between perceived visual quality and cultural symbolism. At Daerim Central Market, Seoul, Symbolism showed the strongest correlation with Trendiness (r = .643, p < .001), indicating a close association between cultural symbolism and online visibility. In Incheon Chinatown, Trendiness showed the strongest correlation with Social Recognition and Sharing (r = .532, p < .001), indicating a close association between online visibility and the extent to which the district was recognized and shared through social networks. No correlation among the variables was extremely high, suggesting no obvious variable redundancy; the VIF results from the subsequent linear mixed-effects models provided a further assessment of multicollinearity. As a supplementary diagnostic, Harman’s single-factor test showed that the first unrotated factor accounted for 39.6% of the total variance. Although this finding suggests that no single factor dominated the covariance structure, it does not exclude common method bias.

### Cross-site comparative analysis

Before fitting the path models, we used repeated-measures ANOVA to examine differences in the five perceived design factors across the three study sites. Mauchly’s test indicated that the sphericity assumption was met for Convenience (W = .963, p = .073), Social Recognition and Sharing (W = .982, p = .289), and Trendiness (W = .973, p = .150). In contrast, this assumption was violated for Aesthetic (W = .870, p < .001) and Symbolism (W = .918, p = .002). We therefore reported Greenhouse–Geisser-corrected degrees of freedom and p values for Aesthetic (ε = .885) and Symbolism (ε = .924).

As shown in Table 5, significant site effects were observed for all five perceived design factors, with the largest effect sizes for Symbolism (partial η² = .156) and Aesthetic (partial η^2^ = .150). Bonferroni-adjusted pairwise comparisons showed that Incheon Chinatown was rated significantly higher than the Ansan Multicultural Special Zone on all five factors and higher than Daerim Central Market, Seoul, on CO, AE, SY, and TR. No significant differences were observed between the Ansan Multicultural Special Zone and Daerim Central Market, Seoul.

**Table 5:** Repeated-measures ANOVA comparing perceived design factor scores across the three multicultural commercial streets (N = 142).

Factor	AnsanM ± SD	SeoulM ± SD	IncheonM ± SD	F (df)	*p*	η²ₚ
CO	3.69 ± 0.97^a^	3.71 ± 1.04^a^	3.99 ± 0.84^b^	7.736(2, 282)	.001	.052
SO	3.46 ± 1.04^a^	3.66 ± 1.03^ab^	3.72 ± 0.99^b^	4.498(2, 282)	.012	.031
AE	3.30 ± 1.11^a^	3.35 ± 1.09^a^	3.96 ± 0.96^b^	24.960(1.77, 249.63)	< .001	.150
SY	3.44 ± 1.04^a^	3.35 ± 0.95^a^	3.99 ± 0.83^b^	25.973(1.85, 260.60)	< .001	.156
TR	3.33 ± 1.10^a^	3.47 ± 0.99^a^	3.92 ± 0.89^b^	18.971(2, 282)	< .001	.119

Note: Means sharing the same superscript letter did not differ significantly in Bonferroni-adjusted pairwise comparisons. Greenhouse–Geisser-corrected degrees of freedom are reported for AE and SY.

### Unified path model results

*Model Fit Results*

Table 6 shows that all three models had 3 degrees of freedom and were therefore not fully saturated. The Ansan model showed good overall fit: χ²(3) = 0.653, p = .884, CFI = 1.000, TLI = 1.032, RMSEA = .000, 90% CI (.000, .067), and SRMR = .013. The χ² test was nonsignificant for the Seoul model, χ²(3) = 6.587, p = .086; CFI = .985, TLI = .951, and SRMR = .041 met commonly used reference criteria. However, RMSEA = .092, 90% CI (.000, .189), exceeded the commonly used reference value of .08. For the Incheon model, CFI = .970 and SRMR = .047 also met commonly used reference criteria. However, the χ² test was significant, χ²(3) = 8.793, p = .032; TLI = .898 was slightly below .90; and RMSEA = .117, 90% CI (.030, .211), was relatively high. Together, these indices indicated weaker overall fit than for the Ansan and Seoul models.

**Table 6:** Fit indices of the unified path models across the three study sites.

Site	χ²(df)	*p*	CFI	TLI	RMSEA (90% CI)	SRMR
Ansan	0.653(3)	.884	1.000	1.032	.000 (.000–.067)	.013
Seoul	6.587(3)	.086	.985	.951	.092 (.000–.189)	.041
Incheon	8.793(3)	.032	.970	.898	.117 (.030–.211)	.047

Thus, all three models showed acceptable fit according to CFI and SRMR; however, the relatively high RMSEA values for the Seoul and Incheon models warrant cautious interpretation. Because RMSEA may overestimate model misfit when degrees of freedom are low [39], we did not evaluate model fit using RMSEA alone. Instead, χ², CFI, TLI, SRMR, and the theoretical consistency of the path coefficients were evaluated jointly.

#### Site-specific path analysis results.

Table 7 presents the path coefficients for the unified path models at the three study sites, including the standardized path coefficient β, unstandardized estimate B, standard error, t value, and p value. Fig 4 presents these results.

**Table 7:** Path coefficients of the unified path models for the three study sites.

Site	Path	β	B	SE	t	*p*	Result
Ansan	AE → SY	.645	.601	.060	10.010	< .001	Significant
	AE → TR	.412	.407	.079	5.162	< .001	Significant
	SY → TR	.348	.369	.084	4.363	< .001	Significant
	SY→SO	.175	.175	.095	1.837	.066	Not significant
	TR→SO	.320	.303	.090	3.364	.001	Significant
	AE → CO	.431	.379	.064	5.945	< .001	Significant
	SO→CO	.269	.253	.068	3.702	< .001	Significant
Seoul	AE → SY	.571	.501	.061	8.249	< .001	Significant
	AE → TR	.303	.276	.067	4.085	< .001	Significant
	SY → TR	.470	.486	.077	6.329	< .001	Significant
	SY→SO	.273	.295	.101	2.914	.004	Significant
	TR→SO	.303	.316	.098	3.229	.001	Significant
	AE → CO	.236	.225	.070	3.228	.001	Significant
	SO→CO	.453	.456	.074	6.203	< .001	Significant
Incheon	AE → SY	.515	.443	.062	7.126	< .001	Significant
	AE → TR	.182	.168	.078	2.154	.031	Significant
	SY → TR	.395	.424	.091	4.681	< .001	Significant
	SY→SO	.317	.376	.092	4.104	< .001	Significant
	TR→SO	.377	.417	.085	4.888	< .001	Significant
	AE → CO	.386	.334	.067	5.005	< .001	Significant
	SO→CO	.206	.175	.065	2.673	.008	Significant

**Fig 4:**
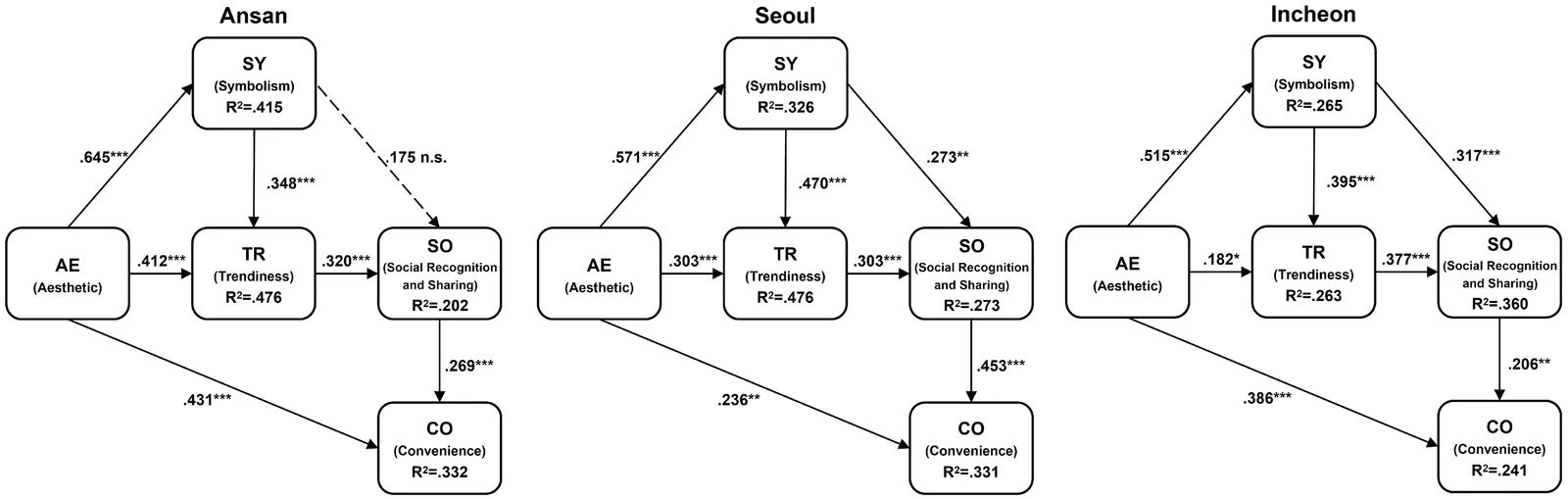
Site-specific path models for the three study sites. Values are standardized coefficients (β); R² indicates explained variance. n.s., nonsignificant; *p < .05, **p < .01, ***p < .001.

In the Ansan Multicultural Special Zone model, six of the seven paths were statistically significant, with SY→SO as the only exception. AE → SY was the strongest path in the Ansan model (β = .645, p < .001), and AE → TR (β = .412, p < .001) and AE → CO (β = .431, p < .001) were also significant. By contrast, SY→SO was not statistically significant (β = .175, p = .066), indicating that cultural Symbolism was not directly associated with ratings of Social Recognition and Sharing in the Ansan model. The model explained 41.5%, 47.6%, 20.2%, and 33.2% of the variance in SY, TR, SO, and CO, respectively.

All seven paths were significant in the Daerim Central Market, Seoul, model. AE → SY (β = .571, p < .001) and SY → TR (β = .470, p < .001) showed relatively strong associations. SO→CO was the most prominent path in the Seoul model (β = .453, p < .001), suggesting that Social Recognition and Sharing was especially strongly associated with Convenience ratings at this site. The model explained 32.6%, 47.6%, 27.3%, and 33.1% of the variance in SY, TR, SO, and CO, respectively.

All seven paths were significant in the Incheon Chinatown model. AE → SY remained relatively strong (β = .515, p < .001), whereas AE → TR was weaker but still significant (β = .182, p = .031). SY → TR, SY→SO, and TR→SO were also significant, reflecting a relatively continuous pattern of associations among Symbolism, Trendiness, and Social Recognition and Sharing in the Incheon model. The model explained 26.5%, 26.3%, 36.0%, and 24.1% of the variance in SY, TR, SO, and CO, respectively.

Across the three site-specific models, AE → SY was consistently prominent, while the principal site-specific features were the nonsignificant SY→SO path in Ansan and the relatively strong SO→CO path in Seoul.

### Cross-site path differences

S1 Table provides the variance components, ICC values, and collinearity diagnostics for the four linear mixed-effects models. The maximum VIF values were 2.938 for the SY model, 5.025 for the TR model, 4.358 for the SO model, and 3.180 for the CO model, with detailed VIF values reported for individual fixed-effect terms. Cross-site interaction tests are reported in Table 8.

**Table 8:** Site interaction tests for the seven paths.

Path	Interaction term	F(df1, df2)	*p*	Unadjusted result
AE → SY	AE × Site	F(2, 396) = 1.71	.183	p ≥ .05
AE → TR	AE × Site	F(2, 417) = 2.42	.090	p ≥ .05
SY → TR	SY × Site	F(2, 417) = 0.52	.597	p ≥ .05
SY→SO	SY × Site	F(2, 378) = 0.26	.771	p ≥ .05
TR→SO	TR × Site	F(2, 377) = 0.55	.579	p ≥ .05
AE → CO	AE × Site	F(2, 356) = 2.12	.122	p ≥ .05
SO→CO	SO × Site	F(2, 341) = 4.58	.011	p < .05

Note: The p values for the seven prespecified interaction tests are unadjusted for multiplicity.

Among the seven prespecified interaction tests, SO × Site was the only interaction with an unadjusted p value below .05, F(2, 341) = 4.58, p = .011; the other six interaction tests had unadjusted p values above .05. We therefore conducted a simple slope analysis and pairwise slope comparisons for SO→CO. Table 9 presents the results. Because Table 9 reports estimates from the pooled linear mixed-effects model, its unstandardized coefficients cannot be directly compared with those from the site-specific path models in Table 7.

**Table 9:** Simple slopes and pairwise slope comparisons for the SO→CO path.

Analysis	Site / Comparison	B	SE	df	t	*p*	Result
Simple slope	Ansan	.227	.063	390	3.59	< .001	Significant
	Seoul	.434	.066	390	6.63	< .001	Significant
	Incheon	.178	.071	385	2.51	.013	Significant
Pairwise comparison	Ansan − Seoul	−.207	.087	332	−2.39	.035	Significant
	Ansan − Incheon	.049	.092	350	0.53	.594	Not significant
	Seoul − Incheon	.257	.093	341	2.77	.018	Significant

Note: p values for the simple slopes are unadjusted. p values for the three pairwise slope comparisons are Holm-adjusted.

Simple slope analysis showed that the SO→CO path was positive and statistically significant at all three study sites. The slope was greatest for Daerim Central Market, Seoul (B = 0.434, SE = 0.066, p < .001), followed by the Ansan Multicultural Special Zone (B = 0.227, SE = 0.063, p < .001) and Incheon Chinatown (B = 0.178, SE = 0.071, p = .013). Holm-adjusted pairwise slope comparisons showed that the SO→CO slope for Daerim Central Market, Seoul, was significantly greater than those for the Ansan Multicultural Special Zone and Incheon Chinatown, whereas slopes did not differ significantly between the Ansan Multicultural Special Zone and Incheon Chinatown. Thus, Social Recognition and Sharing showed the strongest association with Convenience at Daerim Central Market, Seoul.

To examine whether this interaction pattern persisted after covariate adjustment, we conducted an additional robustness test. After adjustment for gender, age, education, and survey mode, the SO × Site interaction remained significant, F(2, 336) = 4.83, p = .0086, indicating that this interaction pattern persisted after covariate adjustment. S2 Table presents the detailed results.

## Discussion and Conclusions

Overall, the three multicultural commercial streets showed a broadly similar structure of perceived design evaluation, while cross-site variation was most evident for the SO→CO association. This shared structure provides the context for interpreting how the physical and visual environment, cultural symbolism, online visibility, social recognition and sharing, and functional convenience were related across the three district types.

## Discussion

The physical and visual environment was consistently associated with evaluations of cultural symbolism. The AE → SY path was significant at all three sites and was among the most stable shared paths. Visual features—including streetscapes, building façades, colors, signage, and the overall spatial atmosphere—were associated with users’ judgments not only of a district’s appearance but also of its cultural symbolism and place distinctiveness. This finding aligns with servicescape and streetscape perception research linking the physical environment to spatial evaluation [19–21] and with place-image research on the relationships among spatial symbols, place recognition, and image formation [12,13]. AE → CO was also significant at all three sites, indicating an association between a more favorable visual environment, clearer spatial recognition, and more positive perceptions of convenience. Thus, the cultural meaning of multicultural commercial streets is tied not only to ethnic food, cultural symbols, and historical narratives but also to the physical environment that users perceive and recognize.

Cultural representation, online visibility, and social recognition and sharing formed a relatively continuous sequence of associations. The SY → TR and TR→SO paths were significant at all three sites. Users who perceived stronger cultural Symbolism in a district were also more likely to rate it as having online visibility and communicative potential, while evaluations of Trendiness were associated with evaluations of social recognition and sharing. This pattern is consistent with research on destination image, city branding, and user-generated content that describes relationships among symbolic recognition, online communication, and place perception [12–16]. The pattern further suggests that the appeal of multicultural commercial streets lies not simply in displaying cultural differences, but in whether cultural characteristics are sufficiently distinctive to be recognized and shared through social networking, thereby contributing to social recognition and sharing.

The site-specific models yielded different significance patterns for the SY→SO path: the path was significant in the models for Daerim Central Market in Seoul and Incheon Chinatown but not in the Ansan Multicultural Special Zone model. However, the SY × Site interaction was not significant, so the strength of the SY→SO path did not differ significantly across the three sites. The Ansan estimate should therefore be interpreted as a site-specific nonsignificant association, not as evidence of a statistically confirmed cross-site difference. Although Ansan has a clear policy identity as a designated multicultural village special zone [9,25], cultural Symbolism showed no statistically significant direct association with evaluations of social recognition and sharing in the Ansan model. Future design and management in policy-designated multicultural districts could examine more closely how cultural symbols relate to recognition and sharing through social networking.

The SO→CO path showed evidence that the association between social recognition and sharing and perceived Convenience varied across sites. Although the SO→CO path was significant at all three sites, its cross-site variation was more evident than that of the other prespecified paths, with the strongest association at Daerim Central Market, Seoul. In this everyday transnational commercial district, the stronger SO→CO association may partly reflect the practical role of place information encountered through social networks in helping users become more familiar with shops, activities, and access conditions, thereby supporting more favorable perceptions of Convenience. This interpretation accords with descriptions of Daerim-dong as a commercial and everyday living space for Korean Chinese immigrants [26] and with evidence that social media and user-generated content contribute to place-information searches and destination-image formation [14–16]. The SO→CO associations were weaker in Ansan and Incheon, suggesting that the extent to which social recognition and sharing is associated with perceived Convenience varies by district type and pattern of use. The supplementary robustness test provided further support for this site-dependent pattern.

Repeated-measures ANOVA also showed that Incheon Chinatown, with its history as a tourism district and well-defined cultural image, received higher mean ratings on most perceived design factors. This interpretation accords with earlier descriptions of Incheon Chinatown as a symbolic landscape [28] and with broader tourism research showing how Chinatown identities are constructed and communicated through tourism discourse [29]. The path analysis and linear mixed-effects models, however, showed that mean-rating differences alone did not fully characterize evaluations of multicultural commercial streets. Except for SO→CO, the other six cross-site interaction tests did not show comparable evidence of site variation, providing evidence of some stability in the unified path structure proposed here. Comparative research should therefore consider not only site-level differences in ratings but also the structural associations among the perceived design factors.

Taken together, the seven theoretical paths received relatively consistent empirical support across the three multicultural commercial streets. AE → SY, AE → TR, AE → CO, SY → TR, and TR→SO were significant at all three sites and showed no significant cross-site differences, documenting recurrent directional associations among the physical environment, cultural symbolism, digital communication, social recognition and sharing, and functional convenience. The nonsignificant SY→SO estimate in Ansan and the stronger SO→CO path in Seoul nevertheless suggest that the expression of this shared structure may vary by site context, with the clearest evidence of cross-site variation observed for SO→CO.

### Theoretical and practical implications

This study contributes theoretically by integrating perspectives from urban design, servicescapes, destination image, and digital communication into the evaluation of multicultural commercial streets [12–16,19–21] and by organizing the five perceived design factors within a testable observed-variable path model. The model treats these evaluations not as isolated judgments of individual design elements but as directional associations among visual quality, cultural representation, online visibility, social recognition and sharing, and functional convenience. This perspective extends evaluation beyond comparisons of individual elements to an analysis of structural relationships.

Methodologically, the combination of site-specific path models and linear mixed-effects models allowed the path structure within each district and cross-site differences in the same paths to be evaluated within a single analytical strategy [33–36]. Unlike simple comparisons of path-coefficient magnitudes, this approach distinguishes shared path structures from site-specific features and accommodates data from participants who repeatedly evaluated multiple sites.

In practical terms, the findings point to an integrated design-management approach in which cultural representation, physical and visual quality, online visibility, social recognition and sharing, and functional convenience are addressed as interconnected dimensions of multicultural commercial-street regeneration. This interpretation is consistent with research on servicescapes, place image, online communication, and ethnically diverse urban environments [12–16,19–21,40–43]. The recurring patterns of AE → SY, AE → TR, AE → CO, SY → TR, and TR→SO across the three sites support coordinating streetscape and façade quality, culturally distinctive shopfronts and signs, pedestrian and commercial information, and the presentation of place information across physical and digital channels. Such coordination shifts attention from simply adding symbolic decoration to improving how existing cultural features, businesses, routes, and local narratives are recognized, interpreted, located, and shared.

The site-specific results suggest different design-management emphases for the three district types. In the Ansan Multicultural Special Zone, where SY→SO was not significant in the site-specific model, greater attention to the legibility and communicability of existing cultural symbols may strengthen their connection with social recognition and sharing through social networks. Multilingual cultural-route maps, interpretive information associated with particular streets and businesses, coordinated visual-information systems, and locally produced digital content represent possible ways of making cultural features more identifiable and communicable. Research on participatory design and place-based storytelling further indicates that involving local merchants and residents can broaden the range of identities and everyday experiences represented in such materials [41,42,44]. At Daerim Central Market in Seoul, where SO→CO showed the strongest association, the findings highlight the value of making place information shared through social networks readily usable for navigation and access to everyday services. Multilingual entrance maps, consistent shop-location information, pedestrian directions from nearby transit access points, and regularly updated online directories could make such information more accessible to both regular and unfamiliar users. In Incheon Chinatown, where the five perceived design factors received comparatively favorable evaluations and SY, TR, and SO showed relatively continuous associations, the results place greater emphasis on sustaining and interpreting the existing cultural and historical image than on extensive physical redesign. Coordinated heritage information, route-based interpretation, and digital storytelling linked to particular streets, buildings, and businesses could reinforce the continuity between cultural symbolism, online visibility, and social recognition while preserving the district’s everyday commercial character.

More broadly, the five perceived design factors may serve as a structured framework for diagnosing and monitoring design conditions in other multicultural commercial districts. When combined with on-site spatial assessment, the framework can help identify whether greater attention is needed in visual quality, cultural legibility, online visibility, social recognition and sharing, or everyday convenience. This approach is also consistent with recent guidance emphasizing place-sensitive and inclusive planning in migration-related urban contexts [45]. The resulting diagnosis can inform the prioritization of spatial, informational, and communication-oriented measures rather than approaching multicultural regeneration primarily as a uniform branding exercise. Periodic reassessment using the same indicators can then document changes in user perceptions and inform subsequent adjustments to design and management strategies.

### Limitations and future research

Several limitations should be noted. First, each of the five perceived design factors was measured with a single item. Although this choice reduced respondent burden and was suitable for repeated evaluations of multiple sites [30,31], single-item measures cannot fully represent the specific meaning of each perceived design factor or support conventional tests of internal consistency. Future research could develop and validate multi-item scales to strengthen measurement stability and construct interpretability. The SO item, in particular, assessed how well an area was known and shared through social networking. It therefore primarily captured social recognition and sharing rather than direct face-to-face interaction or observed street sociability.

Second, the theory-guided recursive path model was developed from cross-sectional survey data. The findings should therefore be interpreted as directional associations within a prespecified theoretical structure, not as causal relationships in the strict sense [33,34]. Other theoretically plausible equivalent models or reverse-path models may fit the data similarly. Longitudinal surveys, on-site behavioral observations, interviews, and experimental designs could help future research examine the temporal ordering and behavioral basis of these path relationships.

Third, because the same participants evaluated the three sites consecutively, repeated use of the same item structure may have introduced anchoring effects, order effects, fatigue effects, or common method bias. Harman’s single-factor test provided only a preliminary diagnostic, and participant-level random intercepts in the linear mixed-effects models addressed within-person correlations; neither approach can fully remove bias associated with a common measurement source and consecutive responses. Online participants may also have differed across sites in visit frequency, recency of experience, or familiarity, which may have reduced evaluation accuracy and, to some extent, increased correlations among the variables. Future research could randomize or counterbalance site order, record the frequency and recency of visits to each site, and use time-separated measurements or a larger proportion of on-site surveys to limit these potential biases.

Fourth, only three multicultural commercial streets in South Korea were examined, and the sample was restricted to participants aged 18–40 years. The findings should therefore be generalized cautiously to other countries, other types of multicultural districts, and broader age groups. Future research could include a wider range of sites and participants and compare design-evaluation structures across cities, countries, immigrant communities, and age groups.

Fifth, the analysis focused primarily on users’ subjective evaluations and did not fully incorporate objective spatial indicators, including street morphology, shop density, pedestrian accessibility, public space configuration, and the intensity of social activities. Combining questionnaire data with space syntax, GIS, behavioral observations, or social media data could provide a more comprehensive account of the spatial mechanisms of multicultural commercial streets.

Sixth, the sample included both on-site and online respondents, but its composition was not fully balanced between the two survey modes. Although survey mode was included as a control variable in the robustness test of the SO→CO path, future research could balance the two samples more closely or systematically examine whether survey mode is associated with differences in evaluations and path relationships.

## Conclusions

Comparative analysis of the Ansan Multicultural Special Zone, Daerim Central Market, Seoul, and Incheon Chinatown provided empirical support for an observed-variable path model of design evaluation in multicultural commercial streets. Across the three sites, the results identified a stable association between the physical and visual environment and the recognition of cultural symbolism, a sequence linking cultural symbolism and online visibility to social recognition and sharing, and evidence that the association between social recognition and sharing and Convenience may vary by site.

AE → SY, AE → TR, AE → CO, SY → TR, and TR→SO were significant across all three sites, and showed broadly consistent patterns across sites. Among them, AE → SY, SY → TR, and TR→SO describe a possible sequence of associations from the visual environment to cultural recognition and then to online communication and social recognition and sharing. AE → TR and AE → CO represent additional direct associations between visual quality and online visibility and between visual quality and functional convenience, respectively. The SY→SO path was not significant in the Ansan site-specific model, although its cross-site interaction was also nonsignificant. Within the Ansan model, cultural Symbolism was therefore not directly associated with evaluations of social recognition and sharing. The stronger SO→CO path in the Seoul model remained robust after adjustment for demographic characteristics and survey mode, documenting a closer association of social recognition and sharing with perceptions of Convenience in an everyday transnational commercial district.

The design value of multicultural commercial streets lies not in cultural diversity alone but also in the connections among the physical environment, cultural symbolism, digital communication, social recognition and sharing, and functional convenience. Future spatial design should extend beyond the display of cultural differences to foster urban commercial environments that are recognizable, communicable, shareable, and sustainable in everyday use.

## Supporting information

S1 Text Complete questionnaire used in the study. (DOCX)

S1 Data Dataset used for the analyses reported in this study. (XLS)

S1 Table Diagnostics and detailed VIF values for the linear mixed-effects models. (DOCX)

S2 Table Robustness check for the SO→CO interaction after controlling for demographic characteristics and survey mode. (DOCX)

## References

[1] Florida R. The flight of the creative class: The new global competition for talent. Liberal Educ. 2006;92(3):22–9.

[2] Sohrabi H. A Durkheimian critique of contemporary multiculturalism. Ethnic and Racial Studies. 2019;42(8):1283–304. doi: 10.1080/01419870.2019.1585898

[3] Hassen I, Giovanardi M. The difference of ‘being diverse’: City branding and multiculturalism in the ‘Leicester Model’. Cities. 2018;80:45–52. doi: 10.1016/j.cities.2017.06.019

[4] Niebuhr A. Migration and innovation: Does cultural diversity matter for regional R&D activity?. Papers in Regional Science. 2010;89(3):563–86. doi: 10.1111/j.1435-5957.2009.00271.x

[5] Fainstein SS. The just city. Int J Urban Sci. 2013;18(1):1–18. doi: 10.1080/12265934.2013.834643

[6] Hunter WC. The social construction of tourism online destination image: A comparative semiotic analysis of the visual representation of Seoul. Tourism Management. 2016;54:221–9. doi: 10.1016/j.tourman.2015.11.012

[7] Kim AE. Global migration and South Korea: foreign workers, foreign brides and the making of a multicultural society. Ethnic and Racial Studies. 2009;32(1):70–92. doi: 10.1080/01419870802044197

[8] Ansan city status [Internet]. Ansan, Ansan City; 2026 [cited 2026 Aug 10]. Available from: https://www.ansan.go.kr/www/common/cntnts/selectContents.do?cntnts_id=C0001097

[9] Heo K. Changing Multicultural Governances : the case of Ansan Multicultural Village Special Zone. JKARG. 2020;26(2):159–71. doi: 10.26863/jkarg.2020.5.26.2.159

[10] Jun H-J. The reciprocal relationship between social capital and community development in a Korean Chinese enclave: the case of Daerim 2-dong in Seoul. International Journal of Urban Sciences. 2022;27(3):518–42. doi: 10.1080/12265934.2022.2134181

[11] Incheon Chinatown [Internet]. Incheon: Incheon Metropolitan City; [cited 2026 Aug 10]. Available from: https://www.incheon.go.kr/en/EN030115/view?contentid=264513&curPage=2

[12] Echtner CM, Ritchie JRB. The Measurement of Destination Image: An Empirical Assessment. Journal of Travel Research. 1993;31(4):3–13. doi: 10.1177/004728759303100402

[13] Kavaratzis M. From city marketing to city branding: Towards a theoretical framework for developing city brands. Place Brand Public Dipl. 2004;1(1):58–73. doi: 10.1057/palgrave.pb.5990005

[14] Xiang Z, Gretzel U. Role of social media in online travel information search. Tour Manag. 2010;31(2):179–88. doi: 10.1016/j.tourman.2009.02.016

[15] Lam JMS, Ismail H, Lee S. From desktop to destination: User-generated content platforms, co-created online experiences, destination image and satisfaction. J Destin Mark Manag. 2020;18:100490. doi: 10.1016/j.jdmm.2020.100490

[16] Guerreiro MM, Pinto PS, Ramos CMQ, Matos N, Golestaneh H, Sequeira B, et al. The online destination image as portrayed by the user-generated content on social media and its impact on tourists’ engagement. Tour Manag Stud. 2024;20(4):1–15. doi: 10.18089/tms.20240401

[17] Choi S, Song J, Kwon D, Kim BHS. Population decline and public attitudes toward multicultural immigration policies in South Korea. Population Space and Place. 2024;30(7). doi: 10.1002/psp.2788

[18] Park CJ. Rethinking urban citizenship in the post-COVID South Korean context. J Diaspora Stud. 2022;16(1):55–104. doi: 10.22735/JODS.2022.16.1.55

[19] Bitner MJ. Servicescapes: The impact of physical surroundings on customers and employees. J Mark. 1992;56(2):57–71. doi: 10.1177/002224299205600205

[20] Turley LW, Milliman RE. Atmospheric effects on shopping behavior: A review of the experimental evidence. J Bus Res. 2000;49(2):193–211. doi: 10.1016/S0148-2963(99)00010-7

[21] Lee J, Park S. Current design guidelines’ streetscape improvement for visual perception and walkability: A case study of Sejong City, Republic of Korea. Front Archit Res. 2023;12(3):423–43. doi: 10.1016/j.foar.2022.11.002

[22] Mehta V, Bosson JK. Third places and the social life of streets. Environ Behav. 2010;42(6):779–805. doi: 10.1177/0013916509344677

[23] Yin RK. Case study research and applications: Design and methods. 6th ed. Thousand Oaks: Sage; 2018.

[24] Seawright J, Gerring J. Case selection techniques in case study research: A menu of qualitative and quantitative options. Polit Res Q. 2008;61(2):294–308. doi: 10.1177/1065912907313077

[25] Ansan City Foreign Residents Support Headquarters. Ansan Multicultural Village Special Zone: Special zone status [Internet]. Ansan, Ansan City; [cited 2026 Jul 12]. Available from: https://www.ansan.go.kr/global/common/cntnts/selectContents.do?cntnts_id=C0001007

[26] Kim T-S. Center and margin on the margin: A study of the multilayered (Korean) Chinese Migrant Neighborhood in Daerim-dong, South Korea. Geoforum. 2021;120:165–75. doi: 10.1016/j.geoforum.2021.01.027

[27] Cho HJ. Incheon Jung-gu Chinatown Special Zone selected as an excellent regional special zone. Straight News [Internet]. 2020 Dec 28 [cited 2026 Jul 12]. Available from: https://www.straightnews.co.kr/news/articleView.html?idxno=91178

[28] Lee JW, Lou JJ. The ordinary semiotic landscape of an unordinary place: Spatiotemporal disjunctures in Incheon’s Chinatown. Int J Multiling. 2019;16(2):187–203. doi: 10.1080/14790718.2019.1575837

[29] Santos CA, Belhassen Y, Caton K. Reimagining Chinatown: An analysis of tourism discourse. Tour Manag. 2008;29(5):1002–12. doi: 10.1016/j.tourman.2008.01.002

[30] Bergkvist L, Rossiter JR. The predictive validity of multiple-item versus single-item measures of the same constructs. J Mark Res. 2007;44(2):175–84. doi: 10.1509/jmkr.44.2.175

[31] Diamantopoulos A, Sarstedt M, Fuchs C, Wilczynski P, Kaiser S. Guidelines for choosing between multi-item and single-item scales for construct measurement: A predictive validity perspective. J Acad Mark Sci. 2012;40(3):434–49. doi: 10.1007/s11747-011-0300-3

[32] Willis GB. Cognitive interviewing: A tool for improving questionnaire design. Thousand Oaks: Sage; 2005.

[33] Wright S. The method of path coefficients. Ann Math Stat. 1934;5(3):161–215. doi: 10.1214/aoms/1177732676

[34] Kline RB. Principles and practice of structural equation modeling. 4th ed. New York: Guilford Press; 2016.

[35] Lefcheck JS. piecewiseSEM: Piecewise structural equation modelling in R for ecology, evolution, and systematics. Methods Ecol Evol. 2016;7(5):573–9. doi: 10.1111/2041-210X.12512

[36] Laird NM, Ware JH. Random-effects models for longitudinal data. Biometrics. 1982;38(4):963–74. doi: 10.2307/2529876 7168798

[37] O’Brien RM. A caution regarding rules of thumb for variance inflation factors. Qual Quant. 2007;41(5):673–90. doi: 10.1007/s11135-006-9018-6

[38] Podsakoff PM, MacKenzie SB, Lee J-Y, Podsakoff NP. Common method biases in behavioral research: a critical review of the literature and recommended remedies. J Appl Psychol. 2003;88(5):879–903. doi: 10.1037/0021-9010.88.5.879 14516251

[39] Kenny DA, Kaniskan B, McCoach DB. The Performance of RMSEA in Models With Small Degrees of Freedom. Sociol Methods Res. 2014;44(3):486–507. doi: 10.1177/0049124114543236

[40] Lesan M. Assessing the publicness of three streets in ethnically-diverse neighbourhoods. Local Environment. 2024;29(3):366–85. doi: 10.1080/13549839.2024.2309502

[41] Eizenberg E, Jabareen Y, Arviv T, Arussy D. Urban space of recognition: design for ethno-cultural diversity in the German Colony, Haifa. J Urban Design. 2021;27(2):205–24. doi: 10.1080/13574809.2021.1985395

[42] Rishbeth C, Ganji F, Vodicka G. Ethnographic understandings of ethnically diverse neighbourhoods to inform urban design practice. Local Environment. 2017;23(1):36–53. doi: 10.1080/13549839.2017.1385000

[43] Hall S, Datta A. The translocal street: Shop signs and local multi-culture along the Walworth Road, south London. City, Culture and Society. 2010;1(2):69–77. doi: 10.1016/j.ccs.2010.08.001

[44] Hudak KC. Resident stories and digital storytelling for participatory place branding. Place Brand Public Dipl. 2019;15(2):97–108. doi: 10.1057/s41254-019-00117-7

[45] United Nations Human Settlements Programme (UN-Habitat). Mainstreaming migration and displacement in urban planning and public space development: A survey of best practices and future recommendations [Internet]. Nairobi: UN-Habitat; 2024 [cited 2026 Aug 10]. Available from: https://unhabitat.org/mainstreaming-migration-and-displacement-in-urban-planning-and-public-space-development-a-survey-of

