# Supplementary material for: How do perceived design factors relate to user evaluations of multicultural commercial streets? Evidence from three sites in South Korea
Source: PLoS One. 2026 Oct 5;21(10):e0358048. doi: 10.1371/journal.pone.0358048 (PMC13637886; doi:10.1371/journal.pone.0358048)
Supplement: Supplementary file 1 [file pone.0358048.s001.docx]

**S1 QUESTIONNAIRE**

*Beyond Cultural Diversity: A Data-Driven Analysis of Design Factors in Korean Multicultural Commercial Streets*

Dear Sir/Madam,

You are cordially invited to participate in the research survey entitled:

**BEYOND CULTURAL DIVERSITY: A DATA-DRIVEN ANALYSIS OF DESIGN FACTORS IN KOREAN MULTICULTURAL COMMERCIAL STREETS**

This research is conducted by researchers from the Department of Industrial Design, Hanyang University ERICA Campus, South Korea. Participation is completely voluntary. You may withdraw at any time without any negative consequences. The questionnaire takes approximately 5–7 minutes to complete.

**Research Team:**

• Duanduan Liu (Hanyang University, South Korea)

• Fan Xu (Hanyang University, South Korea)

• Taesun Kim (Hanyang University, South Korea)

**Principal Investigator:** Duanduan Liu — Ph.D. Candidate, Department of Industrial Design, ERICA Campus, Hanyang University, Ansan 15588, Korea

**Email:** liuduanduan5@hanyang.ac.kr

**ORCID:** https://orcid.org/0009-0001-1753-2105

Thank you for your time and support.

**Informed Consent**

By proceeding with this questionnaire, you confirm that:

- You have read and understood the information provided above
- You are aged 18 years or above
- Your participation is voluntary and you may withdraw at any time
- You agree to participate in this study

☐ **I agree to participate in this survey**

*(Please check the box above to proceed)*

**Part One: SQ Questions — Survey Items for Sample Characteristics**

1. Gender:

☐ Male	☐ Female

2. Age: _______ years old

3. What is your nationality?

☐ Korean

☐ Chinese

☐ Other (Please specify: _________)

4. What is your highest level of education?

☐ High School	☐ University degree	☐ Graduate degree or above

5. How did you participate in this survey?

☐ On-site	☐ Online

**Part Two: Core Research Questions**

*Instructions: Please rate each design factor on a scale from 1 (Strongly Dissatisfied) to 5 (Strongly Satisfied) based on your personal experience visiting each area. All questions must be answered.*

**Section A: Ansan Multicultural Special Zone (Wongok-dong, Ansan)**

*Please rate the following design factors of Ansan Multicultural Special Zone (Wongok-dong, Ansan) based on your personal experience visiting this area.*

| **A1 Convenience (CO)**: Degree to which the multicultural zone is easy to access and navigate. | | | | | | |
| --- | --- | --- | --- | --- | --- | --- |
|  | **1** | **2** | **3** | **4** | **5** |  |
| *Strongly Dissatisfied* | ☐ | ☐ | ☐ | ☐ | ☐ | *Strongly Satisfied* |

| **A2 Sociability (SO)**: Extent to which the area is known and shared through social networking. | | | | | | |
| --- | --- | --- | --- | --- | --- | --- |
|  | **1** | **2** | **3** | **4** | **5** |  |
| *Strongly Dissatisfied* | ☐ | ☐ | ☐ | ☐ | ☐ | *Strongly Satisfied* |

| **A3 Aesthetic (AE)**: Aesthetic level of street landscapes and architectural design. | | | | | | |
| --- | --- | --- | --- | --- | --- | --- |
|  | **1** | **2** | **3** | **4** | **5** |  |
| *Strongly Dissatisfied* | ☐ | ☐ | ☐ | ☐ | ☐ | *Strongly Satisfied* |

| **A4 Symbolism (SY)**: Presence of buildings or areas with distinct representativeness or symbolic significance. | | | | | | |
| --- | --- | --- | --- | --- | --- | --- |
|  | **1** | **2** | **3** | **4** | **5** |  |
| *Strongly Dissatisfied* | ☐ | ☐ | ☐ | ☐ | ☐ | *Strongly Satisfied* |

| **A5 Trendiness (TR)**: Extent to which the area has become an internet hotspot or viral street. | | | | | | |
| --- | --- | --- | --- | --- | --- | --- |
|  | **1** | **2** | **3** | **4** | **5** |  |
| *Strongly Dissatisfied* | ☐ | ☐ | ☐ | ☐ | ☐ | *Strongly Satisfied* |

**Section B: Seoul Daerim Central Market (Daerim-dong, Yeongdeungpo-gu, Seoul)**

*Please rate the following design factors of Seoul Daerim Central Market (Daerim-dong, Yeongdeungpo-gu, Seoul) based on your personal experience visiting this area.*

| **B1 Convenience (CO)**: Degree to which the multicultural zone is easy to access and navigate. | | | | | | |
| --- | --- | --- | --- | --- | --- | --- |
|  | **1** | **2** | **3** | **4** | **5** |  |
| *Strongly Dissatisfied* | ☐ | ☐ | ☐ | ☐ | ☐ | *Strongly Satisfied* |

| **B2 Sociability (SO)**: Extent to which the area is known and shared through social networking. | | | | | | |
| --- | --- | --- | --- | --- | --- | --- |
|  | **1** | **2** | **3** | **4** | **5** |  |
| *Strongly Dissatisfied* | ☐ | ☐ | ☐ | ☐ | ☐ | *Strongly Satisfied* |

| **B3 Aesthetic (AE)**: Aesthetic level of street landscapes and architectural design. | | | | | | |
| --- | --- | --- | --- | --- | --- | --- |
|  | **1** | **2** | **3** | **4** | **5** |  |
| *Strongly Dissatisfied* | ☐ | ☐ | ☐ | ☐ | ☐ | *Strongly Satisfied* |

| **B4 Symbolism (SY)**: Presence of buildings or areas with distinct representativeness or symbolic significance. | | | | | | |
| --- | --- | --- | --- | --- | --- | --- |
|  | **1** | **2** | **3** | **4** | **5** |  |
| *Strongly Dissatisfied* | ☐ | ☐ | ☐ | ☐ | ☐ | *Strongly Satisfied* |

| **B5 Trendiness (TR)**: Extent to which the area has become an internet hotspot or viral street. | | | | | | |
| --- | --- | --- | --- | --- | --- | --- |
|  | **1** | **2** | **3** | **4** | **5** |  |
| *Strongly Dissatisfied* | ☐ | ☐ | ☐ | ☐ | ☐ | *Strongly Satisfied* |

**Section C: Incheon Chinatown (Chinatown, Jung-gu, Incheon)**

*Please rate the following design factors of Incheon Chinatown (Chinatown, Jung-gu, Incheon) based on your personal experience visiting this area.*

| **C1 Convenience (CO)**: Degree to which the multicultural zone is easy to access and navigate. | | | | | | |
| --- | --- | --- | --- | --- | --- | --- |
|  | **1** | **2** | **3** | **4** | **5** |  |
| *Strongly Dissatisfied* | ☐ | ☐ | ☐ | ☐ | ☐ | *Strongly Satisfied* |

| **C2 Sociability (SO)**: Extent to which the area is known and shared through social networking. | | | | | | |
| --- | --- | --- | --- | --- | --- | --- |
|  | **1** | **2** | **3** | **4** | **5** |  |
| *Strongly Dissatisfied* | ☐ | ☐ | ☐ | ☐ | ☐ | *Strongly Satisfied* |

| **C3 Aesthetic (AE)**: Aesthetic level of street landscapes and architectural design. | | | | | | |
| --- | --- | --- | --- | --- | --- | --- |
|  | **1** | **2** | **3** | **4** | **5** |  |
| *Strongly Dissatisfied* | ☐ | ☐ | ☐ | ☐ | ☐ | *Strongly Satisfied* |

| **C4 Symbolism (SY)**: Presence of buildings or areas with distinct representativeness or symbolic significance. | | | | | | |
| --- | --- | --- | --- | --- | --- | --- |
|  | **1** | **2** | **3** | **4** | **5** |  |
| *Strongly Dissatisfied* | ☐ | ☐ | ☐ | ☐ | ☐ | *Strongly Satisfied* |

| **C5 Trendiness (TR)**: Extent to which the area has become an internet hotspot or viral street. | | | | | | |
| --- | --- | --- | --- | --- | --- | --- |
|  | **1** | **2** | **3** | **4** | **5** |  |
| *Strongly Dissatisfied* | ☐ | ☐ | ☐ | ☐ | ☐ | *Strongly Satisfied* |

**Part Three: Overall Preference**

6. Among the three multicultural areas, which do you consider to have the best overall design quality?

☐ Ansan Multicultural Special Zone

☐ Seoul Daerim Central Market

☐ Incheon Chinatown
