# Supplementary material for: How do perceived design factors relate to user evaluations of multicultural commercial streets? Evidence from three sites in South Korea
Source: PLoS One. 2026 Oct 5;21(10):e0358048. doi: 10.1371/journal.pone.0358048 (PMC13637886; doi:10.1371/journal.pone.0358048)
Supplement: Supplementary file 2 [file pone.0358048.s003.docx]

**S3 Table. Diagnostics and detailed VIF values for the linear mixed-effects models.**

Table A summarizes the model-level variance components, ICC values, and maximum VIFs. Table B reports the VIF values for the individual fixed-effect terms.

**Table A. Model-level diagnostics for the linear mixed-effects models.**

| Model | Dependent variable | Fixed-effect structure | Maximum VIF | Random-intercept variance | Residual variance | ICC |
| --- | --- | --- | --- | --- | --- | --- |
| Model 1 | SY | AE, site, AE × site | 2.938 | 0.0058 | 0.5804 | 0.0098 |
| Model 2 | TR | AE, SY, site, AE × site, SY × site | 5.025 | 0.0000 | 0.5842 | 0.0000 |
| Model 3 | SO | SY, TR, site, SY × site, TR × site | 4.358 | 0.2258 | 0.5397 | 0.2950 |
| Model 4 | CO | AE, SO, site, AE × site, SO × site | 3.180 | 0.1939 | 0.4460 | 0.3030 |

**Table B. VIF values for individual fixed-effect terms.**

| Model | Dependent variable | Fixed-effect term | VIF |
| --- | --- | --- | --- |
| Model 1 | SY | AE_c | 2.938 |
|  |  | Seoul | 1.385 |
|  |  | Incheon | 1.497 |
|  |  | AE_c × Seoul | 1.997 |
|  |  | AE_c × Incheon | 1.985 |
| Model 2 | TR | AE_c | 5.025 |
|  |  | SY_c | 4.650 |
|  |  | Seoul | 1.410 |
|  |  | Incheon | 1.554 |
|  |  | AE_c × Seoul | 3.184 |
|  |  | AE_c × Incheon | 2.999 |
|  |  | SY_c × Seoul | 3.066 |
|  |  | SY_c × Incheon | 2.836 |
| Model 3 | SO | SY_c | 4.358 |
|  |  | TR_c | 4.222 |
|  |  | Seoul | 1.415 |
|  |  | Incheon | 1.542 |
|  |  | SY_c × Seoul | 3.203 |
|  |  | SY_c × Incheon | 2.703 |
|  |  | TR_c × Seoul | 3.027 |
|  |  | TR_c × Incheon | 2.616 |
| Model 4 | CO | AE_c | 3.180 |
|  |  | SO_c | 3.172 |
|  |  | Seoul | 1.400 |
|  |  | Incheon | 1.509 |
|  |  | AE_c × Seoul | 2.230 |
|  |  | AE_c × Incheon | 2.325 |
|  |  | SO_c × Seoul | 2.225 |
|  |  | SO_c × Incheon | 2.233 |
| Note: Continuous predictors were mean-centered before the interaction terms were constructed; the suffix “_c” denotes the corresponding mean-centered variable. Ansan served as the reference category for site. VIFs were calculated from the fixed-effects design matrix of each model. ICC was calculated as random-intercept variance divided by the sum of random-intercept and residual variances. | | | |
