# Supplementary material for: How do perceived design factors relate to user evaluations of multicultural commercial streets? Evidence from three sites in South Korea
Source: PLoS One. 2026 Oct 5;21(10):e0358048. doi: 10.1371/journal.pone.0358048 (PMC13637886; doi:10.1371/journal.pone.0358048)
Supplement: Supplementary file 3 [file pone.0358048.s004.docx]

**S4 Table. Robustness check for the SO→CO interaction after controlling for demographic characteristics and survey mode.**

This table reports the interaction test, controlled simple slopes, and pairwise slope comparisons for the SO→CO path.

| Analysis | Site / comparison | B / Bdiff | SE | df | t / F | *p* | Result |
| --- | --- | --- | --- | --- | --- | --- | --- |
| Type III interaction test | SO × site | — | — | 2, 336 | F = 4.83 | .0086 | Significant |
| Simple slope | Ansan | 0.226 | 0.064 | 385 | t = 3.55 | .0004 | Significant |
|  | Seoul | 0.440 | 0.066 | 384 | t = 6.68 | < .001 | Significant |
|  | Incheon | 0.175 | 0.071 | 381 | t = 2.46 | .0142 | Significant |
| Pairwise comparison | Seoul − Ansan | 0.214 | 0.087 | 328 | t = 2.46 | .0290 | Significant |
|  | Incheon − Ansan | -0.051 | 0.092 | 345 | t = -0.55 | .5841 | Not significant |
|  | Seoul − Incheon | 0.264 | 0.093 | 337 | t = 2.84 | .0144 | Significant |
| Note: The model controlled for gender, age, education, and survey mode. *p* values for the interaction test and simple slopes are unadjusted; pairwise comparisons are Holm-adjusted. | | | | | | | |
